# Exposure to Perfluoroalkyl Substances and Mortality for COVID-19: A Spatial Ecological Analysis in the Veneto Region (Italy)

**DOI:** 10.3390/ijerph18052734

**Published:** 2021-03-08

**Authors:** Dolores Catelan, Annibale Biggeri, Francesca Russo, Dario Gregori, Gisella Pitter, Filippo Da Re, Tony Fletcher, Cristina Canova

**Affiliations:** 1 Department of Statistics, Computer Science, Applications ‘G. Parenti’ (DiSIA), University of Florence, 50134 Firenze, Italy; dolores.catelan@unifi.it (D.C.); abiggeri@ds.unifi.it (A.B.); 2 Regional Directorate of Prevention, Food Safety, Veterinary Public Health, Regione del Veneto, 30123 Venice, Italy; francesca.russo@regione.veneto.it (F.R.); filippo.dare@regione.veneto.it (F.D.R.); 3 Unit of Biostatistics, Epidemiology and Public Health, Department of Department of Cardiac, Thoracic, Vascular Sciences and Public Health, University of Padova, 35131 Padova, Italy; dario.gregori@unipd.it; 4 Screening and Health Impact Assessment Unit, Azienda Zero, Regione del Veneto, 35131 Padova, Italy; gisella.pitter@azero.veneto.it; 5 Department of Public Health, Environments and Society, London School of Hygiene & Tropical Medicine, London WC1H 9SH, UK; tony.fletcher@lshtm.ac.uk

**Keywords:** PFAS, COVID-19 mortality, ecological analysis, epidemiological surveillance, hierarchical Bayesian models

## Abstract

Background: In the context of the COVID-19 pandemic, there is interest in assessing if per- and polyfluoroalkyl substances (PFAS) exposures are associated with any increased risk of COVID-19 or its severity, given the evidence of immunosuppression by some PFAS. The objective of this paper is to evaluate at the ecological level if a large area (Red Zone) of the Veneto Region, where residents were exposed for decades to drinking water contaminated by PFAS, showed higher mortality for COVID-19 than the rest of the region. Methods: We fitted a Bayesian ecological regression model with spatially and not spatially structured random components on COVID-19 mortality at the municipality level (period between 21 February and 15 April 2020). The model included education score, background all-cause mortality (for the years 2015–2019), and an indicator for the Red Zone. The two random components are intended to adjust for potential hidden confounders. Results: The COVID-19 crude mortality rate ratio for the Red Zone was 1.55 (90% Confidence Interval 1.25; 1.92). From the Bayesian ecological regression model adjusted for education level and baseline all-cause mortality, the rate ratio for the Red Zone was 1.60 (90% Credibility Interval 0.94; 2.51). Conclusion: In conclusion, we observed a higher mortality risk for COVID-19 in a population heavily exposed to PFAS, which was possibly explained by PFAS immunosuppression, bioaccumulation in lung tissue, or pre-existing disease being related to PFAS.

## 1. Introduction

Per- and polyfluoroalkyl substances (PFAS) are a group of man-made organic chemicals with both hydrophilic and hydrophobic functionalities. They are persistent environmental contaminants because of their resistance to biodegradation, photooxidation, direct photolysis, and hydrolysis [1]. PFAS have been manufactured since the 1940s and widely used in a variety of consumer and industrial products such as carpeting, clothing, upholstery, food paper wrappings, fire-fighting foams, and in processes such as PTFE polymer production and metal plating [2]. More than four thousands PFAS are classified by OECD; among them, perfluorooctanoic acid (PFOA), perfluorooctanesulfonic acid (PFOS), and perfluorohexanesulfonic acid (PFHxS), which are slowly eliminated by the human body with estimated half-lives ranging between 2.5 and 6 years [3]. Due to their long half-lives and tendency to bioaccumulate, exposure to PFAS will persist for many years, making them a potential hazard to humans, and therefore, they are included in the Candidate List of Substances of Very High Concern for Authorisation under the European chemicals regulation. See also the Stockholm Convention [4].

PFAS have been associated with several health conditions including hepatotoxicity, dyslipidemia, endocrine outcomes, and immunotoxicity outcomes [5]. Human epidemiological studies suggest that exposure to PFOS, and possibly PFOA, adversely affect serum antibody response following vaccination in children [6], prenatal exposures to PFOS and PFOA may lead to increased propensity of infection [7], and adult PFOA exposure may reduce influenza vaccination effectiveness [8]. While there is little evidence for other PFAS, both PFOA and PFOS have been classified as immunotoxic in three recent reviews which concur that the animal evidence is strong but the evidence from epidemiology is much weaker [9]. The National Toxicology Program concluded that PFOA and PFOS are presumed to pose an immune hazard to humans based on a high level of evidence that they suppressed the antibody response from animal studies and a moderate level of evidence from studies in humans [9]. This evaluation was supported by a more recent review that concluded that PFOA and PFOS are immunotoxic with respect to antigen-specific antibody responses [10], while another review considered the epidemiological evidence insufficient to reach a conclusion about a causal relationship between exposure to PFOA and PFOS and immune-related health conditions in humans [11].

The current coronavirus pandemic is leading to significant impacts on the planet, changing our way of life. Although the virus mechanisms of action and pathogenesis are still not completely elucidated, immune system effects are evident, leading, in many cases, to respiratory distress and weakened specific antibody responses, which may be an important contributor to a more severe clinical course of the infection [12]. Therefore, PFAS may have the potential via immunotoxicity to exacerbate COVID-19 respiratory symptoms or more generally the severity of the disease through a direct or indirect mechanism [13].

One study has reported on the interaction between PFAS and COVID-19 severity, comparing serum PFAS levels in hospitalized cases to levels in non-hospitalized cases [14]. Neither PFOA nor PFOS were associated, but one of the PFAS at lower concentrations, perfluorobutanoic acid (PFBA), showed evidence of an association with COVID-19 severity, with the proportion above the limit of detection being higher for the hospitalized cases. PFBA has a short half-life compared to other PFAS, so it has very low serum concentrations in the general population compared to PFAS with a longer half-life, which is frequently below detection. However, a study of PFAS in human organs at autopsy found that PFBA concentrates in the lung [15].

In the present paper, we report an ecological study of mortality in a population living in an Italian PFAS contaminated region. Briefly, residents in a large area of the Veneto Region (North-Eastern Italy) were exposed to high concentrations of a mixture of PFAS, particularly PFOA, via contaminated drinking water from a manufacturing plant active since the late 1960s, until autumn 2013, when water treatment plants were equipped with granular activated carbon filters [16]. Measurements of drinking water samples during 2013 indicated that in addition to PFOA (median 319 ng/L), other PFAS present were PFBA (median 123 ng/L) and perfluorobutane sulfonic acid (PFBS) (median 91 ng/L) followed by perfluoropentanoic acid (PFPeA median 70 ng/L), perfluoro hexanoic acid (PFHxA 52 ng/L), PFOS (median 18 ng/L), perfluoroheptanoic acid (PFHpA median 14 ng/L), and PFHxS (median <10 ng/L) [16]. Serum measurements, conducted between July 2015 and April 2016, indicated that most of the serum PFAS raised in the exposed area was PFOA, but also that serum PFAS were higher for eight other PFAS congeners (PFBA, PFPeA, PFHxA, PFHpA, PFDoA, PFBS, PFHxS, and PFOS), including PFBA than in non-exposed areas. Serum levels in the exposed areas were much higher for PFOA (median 14, 95th percentile 248 ng/mL) than PFBA (median below detection, 95th percentile 0.6 ng/mL), reflecting the more rapid excretion of PFBA [17]. Based on measurements carried out by the Environmental Protection Agency of Veneto Region and on the territory served by contaminated waterworks, 30 municipalities have been labeled as area of maximum exposure (Red Area) (for a population of about 154,000 inhabitants in the year 2020).

The objective of this paper is to evaluate at the ecological level if the geographical distribution of mortality for COVID-19 is associated with PFAS exposure in the Veneto Region. To this purpose, we fitted a hierarchical Bayesian model with spatially structured random components.

## 2. Materials and Methods

### 2.1. Data

COVID-19 mortality data at the municipality level for the period between 21 February and 15 April 2020 have been made available by the Directorate of Prevention, Food Safety, Veterinary Public Health of the Veneto Region. The chosen period covers the first wave of the COVID-19 pandemic in Italy and the same months of the five previous years.

The total number of deaths by municipality was made available by the Ministry of Internal Affairs Italian National Resident Population Demographic Archive (Ministero dell’Interno, Anagrafe Nazionale della Popolazione Residente ANPR) and Italian National Institute of Statistics (ISTAT). The data consist of all causes death counts by age group, gender, and municipality for the Veneto region for the period between 1st January and 30th April of the years 2015–2020.

Population data by municipality, year, sex, and age were downloaded from the demographic statistics database of ISTAT [18].

Socio-economic characteristics including education at the municipality level for the population aged 15–60 were obtained as z-scores from Rosano et al. [19].

The numbers and location of the Nursing Homes (NH) for the Elderly in the municipalities of Veneto Region were obtained from Regione Veneto, Area Sanità e Sociale [20].

The following statistical models were fitted for the baseline mortality and the COVID-19 mortality.

### 2.2. Model for Baseline Mortality

Let assume that the observed number of death in the i-th municipality O_i_ (i = 1, …, 563) follows a Poisson distribution with mean *pop_i_ ×* $\theta_{i}$, where *pop_i_* is the person time at risk and $\theta_{i}$, is the mortality rate. A spatial random effect model is used to account for spatial structured and unstructured terms and stabilize rates estimates toward the local and the general mean. We followed the Besag-York-Mollié (BYM) log-linear model [21] in which:

(1) $$\log\left(\theta_{i}\right)=\alpha +\mu_{i}+\nu_{i}$$

where α represents the intercept, $\mu_{i}$ a spatially structured random term and $\nu_{i}$ a spatially unstructured random term. The term $\mu_{i}$*,* called clustering random term, captures Poisson overdispersion which is spatially structured and shrinks the relative risk towards a local mean. The clustering component $\mu_{i}$ is modelled, conditionally on $\mu_{l\sim i}$ terms (*l~ i* denotes the index *l* assumes all integers from 1 to *n_i_*, the number of adjacent areas to the *i*-th ones) as Normal(${\bar{\mu }}_{i}$*, λ_u_n_i_*) where:

(2) $${\bar{\mu }}_{i}=\sum_{l\sim i}\frac{\mu_{l}}{n_{i}}$$

The term $\nu_{i}$, called the heterogeneity random term, captures the overdispersion which is not spatially structured and stabilizes the relative risk toward the global mean. The *a priori* distribution for the heterogeneity is assumed to be Normal(*0, λ_v_*).

The hyperprior distributions of the precision parameters *λ_v_*, *λ_u_* are assumed to be Gamma (0.5, 0.0005) [22]. The α intercept is assumed a priori improper uniform.

This model is used to estimate the spatial distribution of baseline all-cause mortality. The predicted smoothed mortality rate by this BYM model is used as an estimate of the baseline mortality. The baseline mortality is considered a confounding variable to be included in the model for COVID-19 mortality.

### 2.3. Model for COVID-19 Mortality

Let *Y_i_* be the number of deaths for COVID-19 in the *i*-th municipality (*i* = 1, …, 563). The likelihood is assumed to be Negative Binomial with parameters (*p_i_, r*), where *p_i_* = *r*/(*r* + *η_i_*), and *r* is the number of failures in the terminology of the inverse sampling parameterization [23]. These parameters are a function of *η_i_ = pop_i_ × ξ_i_*, where *ξ_i_* is the COVID-19 mortality rate in the *i*-th municipality. We assume a Negative Binomial likelihood to account for overdispersion due to the high number of municipalities with zero counts.

We assume a log-linear model for *ξ_i_*:

(3) $$\log\left(\xi_{i}\right)=\;\alpha +\mu_{i}+\nu_{i}+\beta^{’}x_{i}+\beta_{pfas}I(\mathrm{RedZone}=1)$$

where α, $\mu_{i}$ and $\nu_{i}$ are, respectively, the intercept, cluster, and heterogeneity terms described in the previous section. In this ecological regression framework, the clustering and heterogeneity random terms account for hidden confounders, which are spatially and not spatially structured [24]. The vector $x_{i}$ (*i* = 1, …, 563 municipalities) consists of the *m* potential confounding variables considered in the model. In particular, we considered the smoothed all-cause mortality rate (from the BYM model), the education level (z-score) as a proxy of socio-economic variables, and the proportion of population aged over than 65 yrs. The exposure variable of interest is specified as a dummy variable I (Red_Zone = 1), which indicates the municipalities that belong to the red zone. The vector ***β*** consists in the *m* regression coefficients, and $\beta_{pfas}$ is the coefficient (log rate ratio) for the municipalities of the Red Zone.

The a priori distributions are as follows: *p_i_* is Beta (1,1), *r* is assumed Gamma (0.1, 0.1); ***β*** and $\beta_{pfas}$ are normally distributed with a large variance. Other priors are assumed as in the BYM model as described above.

All the Bayesian analyses were performed using the OpenBugs software [25].

Bayesian inference is based on posterior distributions. The effect estimates are usually a measure of central tendency (we used the mean), and the uncertainty is expressed by an interval over the posterior distribution (we used an equal-tailed Credibility Interval). The posterior probability of relative risk greater than 1 (Prob RR > 1) represents the certainty with which an effect goes in a particular direction—in our case, this is a risk greater than the null value of one. Roughly, this posterior probability has a correspondence with the frequentist one-sided *p*-value through the formula (1—Posterior probability RR > 1) [26,27].

### 2.4. Sensitivity Analysis

We made a sensitivity analysis considering other covariates as potential confounders: the number of NH per municipality and the capacity of each NH.

## 3. Results

### 3.1. Descriptive Analysis

**Figure 1:**
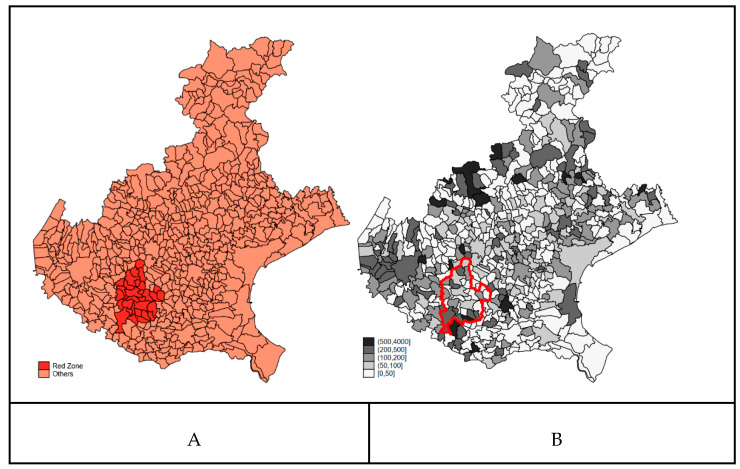
Panel (**A**): Per- and polyfluoroalkyl substances (PFAS) Red Zone in the Veneto Region; Panel (**B**): COVID-19 mortality rate (×100,000) between 21 February and 15 April 2020. Borders of Red Zone are highlighted in red in panel (**B**).

In Figure 1 (Panel A), we reported the municipalities of the Veneto Region, which belong to the Red Zone and the raw mortality rate for COVID-19 for the period between 21 February and 15 April 2020 (Panel B).

The maps of COVID-19 mortality during the first wave of the pandemic (between 21 February and 15 April 2020) (Panel B) showed a geographical pattern with areas at higher risk in the Northern and Western part of the region. The mortality in the municipalities within the Red Zone appeared heterogeneous.

### 3.2. Confounders’ Spatial Distribution

**Figure 2:**
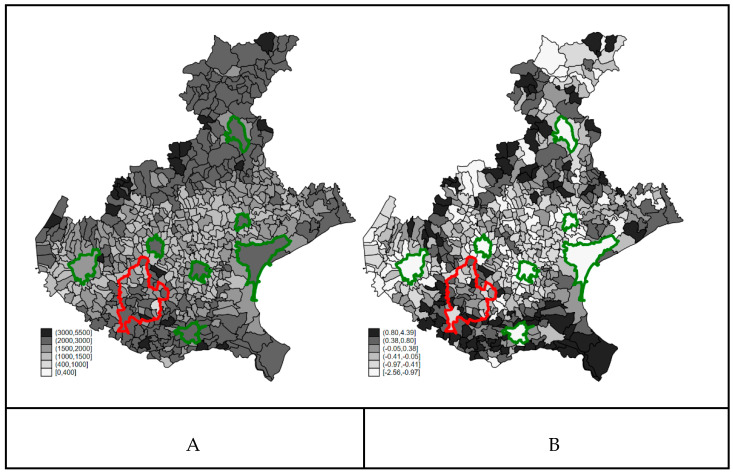
Panel (**A**): Smoothed mortality rates (×100,000) for all cause (January–April 2015–2019); Panel (**B**): Education score (the higher the score, the lower the education level) at the 2011 census. Veneto Region. Borders of the Red Zone are highlighted in red, and those of the main cities of the region are highlighted in green.

The spatial distribution of the smoothed all-cause mortality rate and of the education score (the higher the worse) are shown in Figure 2. Both covariates showed a strong and similar spatial distribution: the higher the all-cause mortality, the lower the education level. The cities of Veneto (green borders in Figure 2) showed higher education levels (lighter shade) and higher mortality rates (darker shade). A certain number of municipalities of the Red Zone presented higher all-cause mortality rates and belong to the fifth or sixth sextile of education score (the higher the score, the lower the education level), with the exception of the southernmost municipalities of the area. The proportion of the population aged older than 65 years was highly collinear with all-cause mortality, and we did not consider further in the analysis.

From a visual inspection of the two figures, some correlation between the map of COVID-19 mortality during the first wave period, between 21 February and 15 April 2020, and the baseline all-cause mortality in the same period over the years 2015–2019 was also suggested: the COVID-19 mortality rate was 18.3 (224 deaths) per 100,000 in the first quartile of baseline all-cause mortality, 17.9 (220) in the second quartile, 25.2 (414) in the third quartile, and 28.6 (237) in the fourth quartile.

### 3.3. Crude Analysis of COVID-19 Mortality in the Red Zone

In Table 1, we show the estimates (and 90% confidence interval CI) of COVID-19 crude mortality rates for the Red Zone and the rest of the Veneto Region. The rate ratio from the data reported in Table 1 is 33.6/21.7 = 1.55 (90% CI 1.25; 1.92), suggesting a strong association between residence in the Red Zone and COVID-19 mortality, comparing to the rest of the Veneto Region.

### 3.4. Bayesian Ecological Regression Analysis

The results of the Bayesian ecological regression model with spatially structured random terms and potential confounders are reported in Table 2. The model included as covariates an indicator for residence in the Red Zone, education level, and baseline all-cause mortality as continuous variables. In the table, we reported the estimated rate ratios (the exponentiated regression coefficients) and the 90% Credibility Intervals. For continuous covariates, rate ratios are expressed per unit change in the Interquartile Range (IQR). For education score, which ranges from –2.6 to + 4.4 with an IQR of 1.23, the estimated rate ratio (the exponential of the regression coefficient) for the IQR is 0.92 (90% Credibility Interval CrI: 0.83; 0.99). During the first wave of the pandemic, the less educated are at lower risk of mortality for COVID-19. For the second covariate included in the model, all-cause mortality 2015–2019, we find a positive association. The estimated rate ratio for unit change of IQR—average annual rate 11.4 SD 3.4 IQR 4.5 per thousand—is 1.04 (90% Credibility Interval CrI: 1.03; 1.05). The adjusted rate ratio for the Red Zone is 1.60 (90% CrI 0.94; 2.51), comparing to the rest of the Veneto Region. The posterior probability RR > 1 is 92.5%.

**Figure 3:**
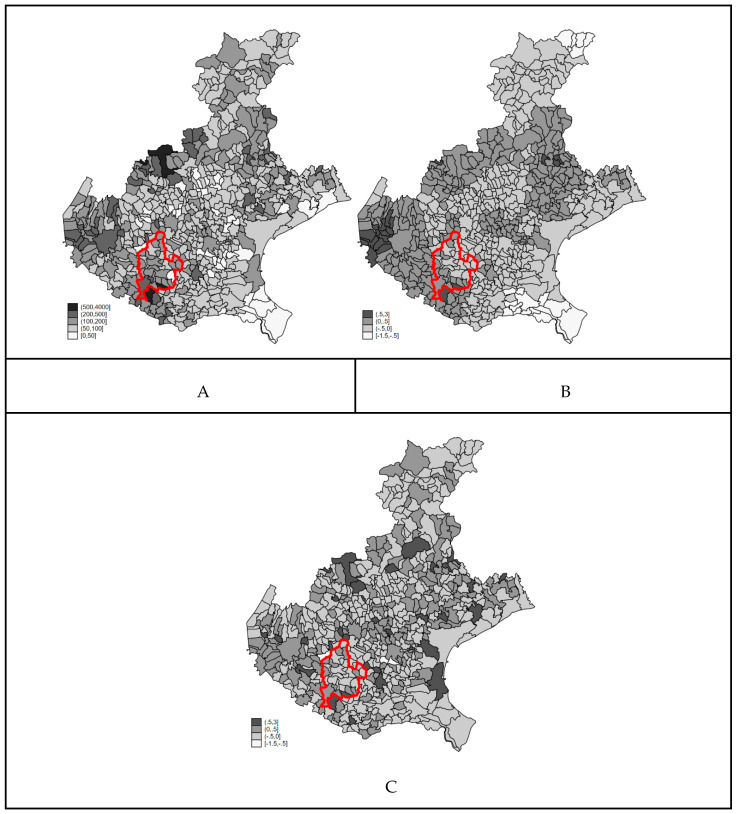
Smoothed mortality rate for COVID-19 (×100,000) (**A**), clustering (**B**), heterogeneity (**C**) random terms from the negative binomial regression models, Veneto Region. Borders of the Red Zone are highlighted in red.

The map of COVID-19 smoothed mortality rates from the Bayesian ecological regression model is reported in Figure 3 (Panel A). The map is very similar to the map of raw COVID-19 mortality rates (Figure 1 Panel B). The spatial distribution of the clustering (B) and heterogeneity (C) random terms might suggest the existence of a long-range trend in the western part of the region, but due to the lack of identifiability of the two random components in the BYM model, we cannot over-interpret this result. Strictly speaking, only the ratio of the standard deviation of the two components can be interpreted. The standard deviation of the clustering term is 0.5243 (±0.213) and that of the heterogeneity term is 0.5861 (±0.14), highlighting that the contribution of the two random components to the explanation of the residual variability is almost the same.

### 3.5. Sensitivity Aanalysis

We made a sensitivity analysis considering the number of NH per municipality, or the capacity of each NH. The rate ratio from the Bayesian ecological regression was 1.08 (90% CrI 1.03; 1.15) for an increase of one NH per municipality. The rate ratio for the Red Zone adjusting for the number of NH did not change significantly (RR 1.58, 90% CrI 0.92; 2.62).

## 4. Discussion

In summary, we observed a higher mortality risk for COVID-19 in a population heavily exposed to PFAS compared to the resident population in the other municipalities of the Veneto region. Regarding the confounders considered in the ecological analysis, we observed that crude all-cause mortality in past years was a good predictor of COVID-19 mortality—the higher the baseline mortality, the higher the deaths for COVID-19—and we observed an inverse relationship between low education level and COVID-19 mortality. In the sensitivity analysis, we also considered NH (care homes for the elderly) as potential confounder and the effect of residence in the Red Zone area was maintained.

Since we conduct the study on all resident population and all COVID-19 deaths occurring in the Veneto Region during the selected time window, a selection bias is excluded. Therefore, we concentrate the discussion on information bias on exposure and confounders; on confounding control and ecological fallacy; and on the interpretation of the association between exposure and outcome. The discussion is structured as follows: (1) consistence of the definition of resident population of the Red Zone as PFAS highly exposed population; (2) consistence of the definition of the observable confounders—NH, baseline mortality, and education; (3) appropriateness of the interpretation of the random effects as hidden confounders in ecological analysis; and (4) interpretation of the ecological association between PFAS exposure and COVID-19 mortality.

### 4.1. Consistence of the Definition of Resident Population of the Red Zone as PFAS Highly Exposed Population

Population sampling of serum PFAS concentrations in the studied population demonstrated that on average, the population living in the Red Zone has much higher PFAS (especially PFOA) than the rest of the Veneto population [16,17]. In terms of daily intake from contaminated water, there is a mixture of PFAS, with PFOA, PFBA, and PFBS showing the highest concentrations. However, in considering these results, the following points should be taken into account. Extensive variation in serum concentrations of PFAS has been reported—PFOA IQR 65.6 ng/mL on 18,345 subjects 14–39 years of age. The 5th percentile was 5.1 [16], and it would be desirable to investigate if there was any difference in COVID-19 rates between those with higher and lower serum PFAS concentrations. Future studies should try to include a quantitative exposure assessment, hopefully at a low spatial resolution. In the literature, there is some information on the PFAS level in individuals residing outside the Red Area [17]. We consider only an indicator variable for the Red Zone, assuming other municipalities of the Veneto Region as not exposed. Moreover, we assume that the relevant exposure refers to the years before interventions in the water supply were made [16]. We also assumed no effect of chronic PFAS exposure on all-cause mortality. The adjusted rate ratio for all-cause mortality 2015–2019 of the Red Zone vs. other municipalities of the Veneto Region was 1.05 (90%CI 0.99; 1.11). This difference in all-cause mortality is small compared to the average increase in COVID-19 mortality for the Red Zone.

### 4.2. Consistence of the Definition of the Observable Confounders—NH, Baseline Mortality, and Education

We did not conduct an age-stratified analysis. We prefer in the ecological regression to include baseline crude mortality as a covariate that takes into account the age structure (percentage of elderly) and baseline frailty of the populations at the municipality level [28]. The percentage of elderly and crude mortality rate are highly collinear. Therefore, we opted to use as a covariate the smoothed Bayesian crude mortality rate. The use of a smoothed Bayesian rate is justified by our belief that the underlying mortality risk—i.e., the population frailty—is spatially structured without important hot spots, with the exceptions of the province capital cities. This pattern can be very efficiently captured by the Besag–York–Mollié random effect model through, respectively, the clustering and heterogeneity components. A more cumbersome analysis would have jointly specified the model on COVID-19 mortality and the model on crude mortality. We estimated the crude mortality rate data from five calendar years, and the uncertainty in the estimates is low. Therefore, by our two-step analysis, we do not expect much modification on the point estimate of the effect of interests, and we do not expect great change in the width of the credible interval [29].

Our results showed that crude all-cause mortality in past years is a good predictor of COVID-19 mortality—the higher the baseline mortality, the higher the deaths for COVID-19. This association is attributable mostly to the effect of the age structure of the municipalities’ populations, which are compared. However, higher mortality could be interpreted as a rough measure of the amount of vulnerable people in given populations [30].

We found an inverse relationship between low education level and COVID-19 mortality. The interpretation of this finding is that communities with a higher percentage of low educated people were less exposed to infection by Sars-Cov-2, at least in the first wave of the pandemic in the Veneto Region. We expected a large variability of COVID-19 mortality rates among municipalities within the Veneto Region. As reported in the literature, “differentiating factors include [a municipality’s] exposure to tradable sectors, its exposure to global value chains, and its specialization” [31]. These factors may be inversely correlated with the percentage of low educated people in the community.

A possible NH (care homes for the elderly) effect cannot be excluded. The highest COVID-19 mortality rates were reported among hosts of Nursing Homes (NH) for the elderly [32]. Therefore, a potential confounder could have been the location of NH. The sensitivity analysis considering the number of NH in the municipality is reassuring. The adjusted rate ratio for the Red Zone did not change significantly (RR 1.58, 90% CrI 0.92; 2.62). However, there is still a potential residual confounding because we had no information of differential COVID-19 mortality by NH. In the lay press, two clusters of COVID-19 deaths were reported in the NHs located in the two Red Zone municipalities with the highest COVID-19 mortality rates.

### 4.3. Appropriateness of the Interpretation of the Random Effects as Hidden Confounders in Ecological Analysis

The Bayesian ecological regression model on COVID-19 mortality includes education score, background mortality, and the indicator for the Red Zone. The two random components—heterogeneity and clustering terms—in the model are intended to adjust for potential hidden confounders [22]. The idea is that the clustering random term—i.e., the spatially structured random term—acts as a flexible stratification that considers for each area the adjacent ones. The other random term—the heterogeneity random term in the BYM model—is intended to adjust for residual rather than spatially structured confounding. An ecological fallacy could still bias our results if effect modification by location is present [33].

### 4.4. Interpretation of the Ecological Association between PFAS Exposure and COVID-19 Mortality

This study is the first investigation of COVID-19 mortality in a population with widespread high exposure to a mixture of PFAS. In looking at mortality, this study cannot distinguish whether PFAS exposure is associated with increased risk of Coronavirus infection, COVID-19 symptoms, or COVID-19 disease severity. The only other study of COVID-19 and PFAS [14] reported an association between PFBA and COVID-19 severity and the fact that the Red Zone population had an unusually high PFBA exposure in the drinking water [16], along with the evidence that PFBA concentrates in lung tissue [15], which suggests PFBA as a potential key exposure. In addition, it is well established that COVID-19 severity and mortality is increased for people with a number of pre-existing conditions [34], so another explanation, other than the immunosuppressive effect of these substances, may simply be that the proportion with some of those pre-existing conditions is higher due to PFAS exposure—even if, at an ecological level, we adjusted for baseline population frailty. PFAS exposure has been associated with a number of conditions including dyslipidemia [35,36] and hypertension [16] also in the studied population. These hypothetical explanatory pathways could be explored with further study of the impact of PFAS on coronavirus infection, on coronavirus vaccine effectiveness, on the pattern or disease in the exposed community, and the characterization of individual PFAS exposure in relation to coronavirus infection and COVID-19. However, given the evidence of immunotoxicity for some PFAS (PFOA and PFOS) and the bioaccumulation of some PFAS in the lung (PFBA), and one other study published showing some evidence of PFBA and COVID-19 severity being associated, a direct effect of PFAS exposure on the risk of COVID-19 is plausible, and assessment of the COVID-19 impact in other populations is needed to see if this finding is replicated.

## 5. Conclusions

In conclusion, we observed a higher mortality risk for COVID-19 in a population heavily exposed to PFAS. If it is not simply a chance association, this might plausibly suggest a general immunosuppressive effect of PFAS, it might be a quite specific effect of PFBA concentrating in the lungs and exacerbating COVID-19 respiratory toxicity, or PFAS might lead to other conditions that predispose people with coronavirus infection to more severe disease, and more work is needed to distinguish these different mechanisms.

## Figures and Tables

**Table 1:** COVID-19 mortality estimated rates and 90% confidence intervals (CI) for the Red Zone and the other municipalities between 15 February and 15 April 2020, Veneto Region.

	Number of Municipalities	COVID-19 Deaths	Population	Rate (×100,000)	90% CI
**Red Zone**	30	63	187,375	33.6	27.3; 41.4
**Others**	533	1032	4,750,548	21.7	26.6; 22.9

**Table 2:** Results of the Bayesian ecological regression model on COVID-19 mortality: adjusted Rate Ratio (RR) and 90% Credibility Interval (CrI) between 15 February and 15 April 2020, Veneto Region.

	RR	90% CrI
General Mortality	1.04	1.03; 1.05
Education Level	0.92	0.83; 0.99
Red Zone	1.60	0.94; 2.51

## Data Availability

The data presented in this study are available on request from the corresponding author.
